# Improving surgical instrument cleaning processes through collaborative efforts between central sterile supply department and operation room

**DOI:** 10.1017/ash.2025.117

**Published:** 2025-09-03

**Authors:** Hsu Xiu-Yue, Lin Li-Hua

**Affiliations:** Taipei Tzu Chi Hospital, Buddhist Tzu Chi Medical Foundation

**Keywords:** precleaning, surgical instrument, infection risk

## Abstract

**Objectives:** The development of medical technology has led to increasingly intricate surgical instruments, varying in types and structures. This complexity has posed challenges in the instrument reprocessing.The Emergency Care Research Institute in the United States has continuously issued alerts regarding the reprocessing of instruments or endoscopes from 2013~2020, It is evident that failure to perform thorough cleaning in accordance with the standards may result in organic debris on the instruments, posing the risk of infection or even death to patients **Methods:** We collected data on 39 visibly soiled instruments reported by the OR from 2021 to October 2023. Through personnel interviews, questionnaires, and identification of types of soiled instruments,we identified key issues using fishbone diagram analysis, the findings as follows (1).only 48% of OR personnel had received precleaning education and training, 75% of respondents cited being too busy for not precleaning (2).CSSD staff demonstrated a cleaning cognitive test success rate of 81%, particularly challenging were instruments with intricate designs, requiring specialized cleaning procedures (3). Failure to provide different cleaning methods depending on the level of residues or difficulty to clean.The following strategies are proposed:(1). Enhanced precleaning education and training for OR staff (2).Use of enzymatic precleaning products to saturate instruments prior to cleaning. 3. Development of a classification system for instruments requiring longer cleaning times. **Results:** Between November 2023 to March 2024, one visibly soiled instrument was reported, marking a significant drop from an incidence rate of 1.15 to 0.20 per month. Cognitive test success rate rose from 81 to 97%. Implementing different methods based on the difficulty of cleaning or the complexity of features, as well as enzymatic precleaning products, were universally adopted in the OR **Conclusion:** Thorough cleaning is a crucial process for effective sterilization. Collaborative efforts between CSSD and OR significantly reduce the possibility of cross-contamination

